# Estimated Energy Expenditures and Energy Intakes of International Female Rugby Sevens Players in Five Days of a Training Camp and Competition Preparation

**DOI:** 10.3390/nu15143192

**Published:** 2023-07-19

**Authors:** Christopher Curtis, Nicola Arjomandkhah, Carlton Cooke, Mayur K. Ranchordas, Mark Russell

**Affiliations:** 1 School of Social and Health Sciences, Leeds Trinity University, Leeds LS18 5HD, UK; ccurtis@unav.es (C.C.); n.arjomandkhah@leedstrinity.ac.uk (N.A.); 2 School of Pharmacy and Nutrition, University of Navarra, 31009 Pamplona, Spain; 3 Carnegie School of Sport, Leeds Beckett University, Headingley Campus, Leeds LS6 3QS, UK; carlton.cooke@leedsbeckett.ac.uk; 4 Academy of Sport and Physical Activity, Health Research Institute and Advanced Wellbeing Research Centre, Sheffield Hallam University, Sheffield S9 3TU, UK; m.ranchordas@shu.ac.uk

**Keywords:** macronutrients, micronutrients, accelerometer, nutrition, female athlete, team sport

## Abstract

To understand the energy balance of international female rugby sevens (R7s) players in applied environments, this study estimated the energy intakes (EI) and total daily estimated energy expenditures (TDEE) during a five-day training camp (TRAIN) and phase of competition preparation (COMP) of equal duration. Tri-axial accelerometer devices were worn throughout both scenarios to estimate TDEE, whereas EI was estimated via self-reported food diaries. Energy deficits of −47% (TDEE_TRAIN_: 14.6 ± 1.6 MJ·day^−1^, EI_TRAIN_: 7.7 ± 0.9 MJ·day^−1^, *p* ≤ 0.001, d = 5.1) and −50% (TDEE_COMP_: 15.5 ± 1.6 MJ·day^−1^, EI_COMP_: 7.7 ± 1.0 MJ·day^−1^, *p* ≤ 0.001, d = 5.7) were observed throughout TRAIN (*n* = 11; age: 25 ± 4 years, height: 170 ± 6 cm, weight: 71 ± 7 kg) and COMP (*n* = 8; age: 25 ± 3 years, height: 172 ± 5 cm, weight: 72 ± 6 kg), respectively. Carbohydrate intakes were below the lower range of sports nutrition recommendations in both TRAIN (−62%; 2.3 ± 0.3 g·kg^−1^ BM, *p* ≤ 0.001) and COMP (−60%; 2.4 ± 0.5 g·kg^−1^ BM, *p* ≤ 0.001). For protein (TRAIN: 1.7 ± 0.4 g·kg^−1^ BM, COMP: 1.5 ± 0.1 g·kg^−1^ BM), intakes met the lower range of recommendations. Fat intake exceeded recommendations of the percentage of total EI (COMP: 39 ± 5%). Accordingly, the dietary strategies of international female R7s players may warrant optimization, as carbohydrate and fat intakes were less than optimal when compared to current performance-based sports nutrition guidelines.

## 1. Introduction

Rugby sevens (R7s) is a contact sport comprising a team of seven players who compete over two 7 min halves. It is most frequently played in tournaments that are contested under nearly identical laws as the 15-person code [1]. Akin to empirical evidence, the pace of R7s exceeds that of the 15-person code, with players reporting ~113–120 m·min^−1^ being covered [1]. Likewise, ~30% of total match distances are covered at speeds ≥ 5 m·s^−1^, while 39% more high velocity (≥4 m·s^−2^) accelerations are performed when players compete in international tournaments [1]; a response that can be repeated up to six times over a typical two- or three-day competition period [2]. Additionally, the frequency of decelerations performed by forward players in specific training scenarios (such as moderate- to high-intensity skill-refining drills) exceeds that of matches [3]. Therefore, to facilitate optimized performance during match-play and the realization of training-induced adaptations, the energy expenditure (EE) and energy intake (EI) of R7s players need to be considered in both training and competition preparation scenarios. This statement may be even more pertinent for female players given the lack of available literature in this population, the anthropometric differences that exist between males and females, and the differing biological responses of female athletes when energy balance (EB) is less than optimal [4,5].

Understanding the EB of athletes enables practitioners to implement appropriate interventions that can improve both health and performance outcomes and allow for the manipulation of body composition [6]. Data relating to the nutritional practices of female athletes are available within a limited number of team sports [7,8,9,10,11], but information concerning the nutritional practices of elite female R7s players is lacking. Although nutritional recommendations for R7s players have been proposed [2], and several studies have investigated micronutrient changes over the course of a competitive season [11,12], such data are specific to male cohorts. Inferring from data from other team sports, female players often do not meet their energy needs [6,7,13] with sub-optimal carbohydrate (CHO) intakes reported relative to sports nutrition recommendations [14,15]. Notably, lower intakes of CHO have been associated with players wishing to promote body composition changes or are attributable to under-reporting in self-reported food diaries (FD) [6].

Acknowledging that alternative methods such as direct calorimetry [16] and doubly labeled water (DLW) [17] may be favorable for the assessment of human metabolic rate and EE, such methods may be impractical in applied environments. DLW has been previously used to assess the energetic demands of collision sports [18,19], but accelerometers provide valid and reliable measures of physical activity [20] in adults under free-living conditions [21] and have been previously used in team sports to estimate EE [7,8,22,23]. Such studies assist in providing insight into the estimated EE of intermittent team sports players. Therefore, with a view to better understanding the EB of international female R7s players in applied environments, the aims of this study were to: a) estimate EE and EI throughout a five-day cycle of training and competition preparation; and b) compare macronutrient and micronutrient intakes of international female R7s players against current published sports nutrition and health guidelines throughout training and competitive scenarios.

## 2. Materials and Methods

Using an observational approach, EB was estimated by the collection of EE (accelerometer-derived) and EI (via FD) data within professional international female R7s players during (a) a training camp (TRAIN) and (b) preparation for an international-standard competition (COMP).

### 2.1. Participants

Twelve international R7s players (International matches: 62 ± 28) competing on behalf of a single National team were initially invited to participate in both phases (TRAIN and COMP) of the study. In TRAIN, 11 female players (age: 25 ± 4 years, height: 170 ± 6 cm, weight: 71 ± 7 kg) participated, whereas in COMP, 8 female players participated (age: 25 ± 3 years, height: 172 ± 5 cm, weight: 72 ± 6 kg); both over a five-day period. The difference in numbers recruited during TRAIN and COMP represents player availability during the training camp (injuries, adapted training, etc.) and variations in squad size selected for travel to the tournament competition. Five players completed both scenarios, with TRAIN being followed by COMP and seven days separating the data collection phases.

The study obtained ethical approval from the School of Social and Health Sciences Ethics Committee at Leeds Trinity University, United Kingdom (Approval Code: SSHS-2018-030), and informed consent was sought from participants prior to study involvement.

### 2.2. Anthropometrics

Participant stature and body mass (BM) were collected at baseline and repeated after the completion of training and competition preparation. Both stature and BM were collected as part of morning player monitoring protocols when participants reported for training and medical screening. In line with established squad protocols, players were asked to report prior to breakfast (following an overnight fast) in a hydrated state in both scenarios. Stature was measured via a portable stadiometer (Seca, Hamburg, Germany) to the nearest 1 mm. BM was measured via calibrated weighing scales (Seca, Hamburg, Germany) to the nearest 0.1 kg.

### 2.3. Energy Expenditure and Total Daily Energy Expenditure

Participants were required to wear a wrist-worn tri-axial accelerometer device (Actigraph wGT3X-BT Monitor, Pensacola, FL, USA) on their dominant wrist to estimate EE. Actigraph wGT3X-BT devices were selected due to their reliability and accuracy at lower intensity expenditures [21,24]; a pertinent point given that the majority of time in this study would represent exposure to lower, as opposed to higher, exercise intensities (i.e., recovery sessions, non-training times, sleep, etc.). In addition, wrist-worn devices have been reported as superior to hip-worn devices when detecting activities involving significant arm movements (such as those involved in R7s) during high-intensity activity [25].

Accelerometer devices were initialized with participant characteristics before the data collection periods, and the sampling frequency was set at 100 Hz. During training, participants were instructed to wear the accelerometer device for five consecutive days during TRAIN. During COMP, participants were instructed to wear the accelerometer device for five consecutive days immediately preceding a competitive match played as part of an international tournament (i.e., match-day minus six through to minus one). In both scenarios, participants were instructed to wear the devices at all times, aside from during periods of exposure to water.

Once the respective data collection periods were completed, participant EE data was combined with corresponding resting energy expenditure (REE) values resulting from BM-derived estimates of REE [26] as per the methods of Drenowatz and Eisenmann [27]. A body mass-based predictive equation, validated in athletes aged 18–35 years, was used to estimate REE [26] as per the methods of Marsh et al. [8]:

REE (Kcal⋅d^−1^): 11.936 × body mass (kg) + 587.728 × Stature (m) − 8.129 × Age (y) + 191.027 × 0 + 29.279

### 2.4. Estimating Dietary Intake

Dietary intake was estimated via five-day FD collected throughout both activity scenarios using validated methods [28]. Participants in both parts of the study were instructed by the principal researcher on how to complete the diaries in a manner that agreed with the practices of the team and required quantification of portion size using household measures, as per the methods of Russell and Pennock [29]. Food diaries were checked for accuracy via a face-to-face consultation with participants upon their completion by the lead researcher and were analyzed post-testing via a nutrition software program (Nutritics v5.04, Education Edition 2018).

### 2.5. Training and Competition Demands

An overview of both training and competition schedules can be seen in Table 1. During TRAIN, there were four prehabilitation (prehab), gym-based (gym), and field-based rugby training (RT) sessions, and one active recovery (AR) session scheduled. During COMP, three prehab sessions, one gym session, four field-based RT sessions, and two AR sessions were scheduled. Briefly, prehab sessions included activities programmed for injury prevention purposes, while RT and gym sessions were focused on primarily technical skills and strength and power development, respectively.

### 2.6. Statistical Analysis

All data were checked for normality using the Shapiro–Wilks test. Within-player differences between the components of EB (estimated TDEE and EI) and BM changes were analyzed using paired samples *t*-tests throughout both TRAIN and COMP. Differences between mean macronutrient intakes and the lower ranges of current sports nutrition recommendations for CHO and PRO and the upper range of current recommendations for FAT were assessed using one-sample *t*-tests as per the methods of Marsh et al. [8]. Differences between mean micronutrient intakes and Scientific Advisory Committee on Nutrition (SACN) daily intake recommendations were assessed using one-sample *t*-tests. For players who completed both trials (*n* = 5), TDEE, EI, EB, and macronutrient data were compared using a paired samples *t*-test. BM data collected at baseline and post-testing were analyzed via two-way ANOVA as a function of trial and time. Mauchly’s test of sphericity was consulted, and the Greenhouse–Geisser correction was applied if the assumption of sphericity was violated. Effect sizes (ES) were calculated in accordance with Cohen’s d ES principles [30]. An alpha level of *p* ≤ 0.05 denoted significance.

## 3. Results

A total of 11 participants were recruited for TRAIN, and 8 participants for the COMP. A total of five participants completed both scenarios. All players completed all training sessions outlined in Table 1.

### 3.1. Training (TRAIN) Scenario

**Figure 1:**
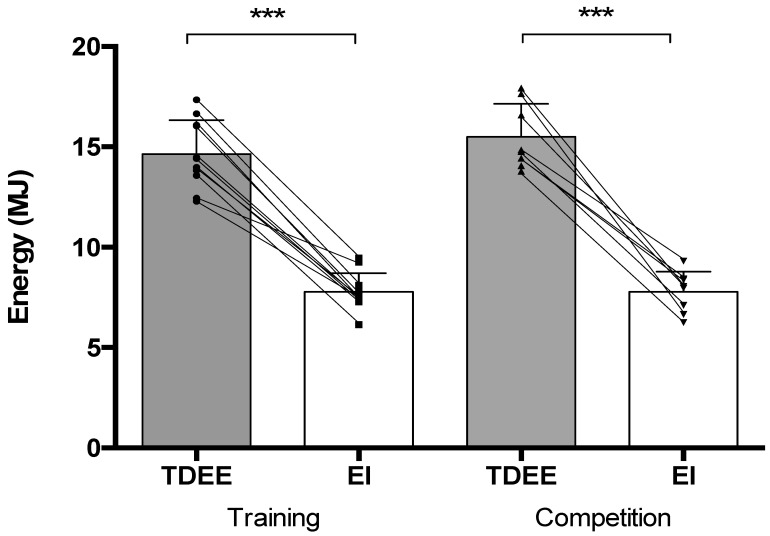
Mean ± standard deviation (denoted by bars) and individual participant (denoted by lines) responses to total daily energy expenditure (TDEE), and energy intake (EI) during training (, *n* = 11) and competition (*n* = 8) assessed over five days. *** indicates within-activity statistical differences between TDEE and EI at *p* ≤ 0.001 level. Individual data points for TDEE and EI are represented by circles and squares, and by up- and down-pointing triangles within the training and competition scenarios, respectively.

**Figure 2:**
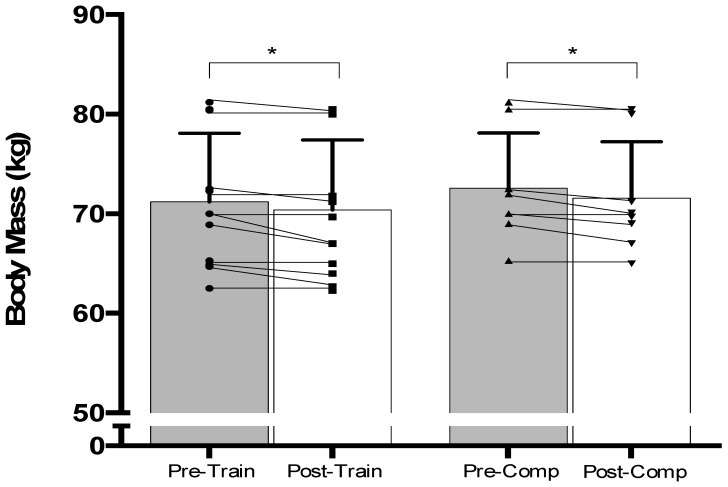
Mean ± standard deviation (denoted by bars) and individual (denoted by lines) body masses (kg) during pre-training and post-training (*n* = 11) and pre-competition and post-competition (*n* = 8) scenarios when assessed at baseline and post-testing. * indicates within-activity statistical differences between at *p* ≤ 0.05 level. Individual data points for body mass are represented by circles and squares, and by up- and down-pointing triangles within the training and competition scenarios, respectively.

Energy deficits of −47% (TDEE_TRAIN_: 14.6 ± 1.6 MJ·day^−1^, EI_TRAIN_: 7.7 ± 0.9 MJ·day^−1^, *p* ≤ 0.001, d = 5.5) were observed (Figure 1). BM losses of 0.8 ± 0.7 kg occurred (pre: 71.2 kg, post: 70.4 kg, *p* ≤ 0.05, d = 0.12; Figure 2). Mean CHO intake in TRAIN (CHO_TRAIN_: 2.3 ± 0.3 g·kg^−1^ BM) was significantly lower than sports nutrition recommendations (−62%: *p* ≤ 0.001, d = 18.2). Mean TRAIN PRO intake (PRO_TRAIN_: 1.7 ± 0.4 g·kg^−1^ BM) exceeded the lower range of sports nutrition recommendations (+42%: *p* = 0.001, d = 1.85), whereas FAT intakes (FAT_TRAIN_: 35 ± 5% total EI) in TRAIN were equal to the upper range of recommended values (+0%: *p* ≥ 0.05, d = 0.01). Mean macronutrient intakes during TRAIN are presented in Table 2.

Mean calcium, iodine, and vitamins A and E in TRAIN were higher than nutrition recommendations (Calcium_TRAIN_: 1047 ± 323 mg, +40%, *p* = 0.003, d = 1.59; Iodine_TRAIN_: 205 ± 83 mg, +38%, *p* = 0.02, d = 1.16; VitaminA_TRAIN_: 857 ± 282 µg, +35%, *p* = 0.009, d = 1.35; VitaminE_TRAIN_: 8.7 ± 3.1 mg, +97%, *p* = 0.001, d = 2.72), whereas potassium and iron in TRAIN were lower than nutrition recommendations (Potassium_TRAIN_: 2980 ± 463 mg, −16%, *p* = 0.003, d = 1.66; Iron_TRAIN_: 11.3 ± 2.3 mg, −27%, *p* = 0.001, d = 2.25; Table 3).

### 3.2. Competition Preparation (COMP) Scenario

Energy deficits of −50% (TDEE_COMP_: 15.5 ± 1.6 MJ·day^−1^, EI_COMP_: 7.7 ± 1.0 MJ·day^−1^, *p* ≤ 0.001, d = 6.2) were observed (Figure 1). BM losses of 1.0 ± 0.7 kg occurred (pre: 72.6 kg, post: 71.6 kg, *p* ≤ 0.05, d = 0.19; Figure 2). Mean CHO intake (CHO_COMP_: 2.4 ± 0.5 g·kg^−1^ BM) did not meet current sports nutrition recommendations (−60%: *p* ≤ 0.001, d = 10.8). Mean COMP PRO intake (PRO_COMP_: 1.5 ± 0.1 g·kg^−1^ BM) was significantly higher than the minimum range of sports nutrition recommendations (+25%: *p* ≤ 0.05, d = 4.5). Mean COMP FAT intakes (FAT_COMP_: 39 ± 5% total EI) exceeded the upper range of sports nutrition recommendations (+11%: *p* ≤ 0.05, d = 2.36).

A summary of mean macronutrient intakes for COMP can be seen in Table 2. Mean selenium and vitamins A, C, and E in COMP were higher than nutrition recommendations (Selenium_COMP_: 77 ± 17 mg, +25%, *p* = 0.02, d = 1.51; VitaminA_COMP_: 1037 ± 269 µg, +53%, *p* = 0.003, d = 2.46; VitaminC_COMP_: 115 ± 69 mg, +97%, *p* = 0.001, d = 1.69; VitaminE_COMP_: 11 ± 2.1 mg, +114%, *p* = 0.0001, d = 5.76; Table 3).

### 3.3. Within-Participant Comparisons between Conditions

For players that completed both scenarios, a non-significant difference for TDEE was observed between conditions (TDEE_TRAIN_: 15.0 ± 1.8 MJ·day^−1^, TDEE_COMP_: 16.0 ± 2.0 MJ·day^−1^, *p* ≥ 0.05, d = 0.59). Similarly, a non-significant difference of −7% for EI was observed between TRAIN and COMP conditions (EI_TRAIN_: 8.0 ± 0.7 MJ·day^−1^, EI_COMP_: 7.5 ± 1.0 MJ·day^−1^, *p* ≥ 0.05, d = 0.77). A 22% difference for EB was observed between conditions (EB_TRAIN_: 7.0 ± 2.4 MJ·day^−1^, EI_COMP_: 8.6 ± 2.1 MJ·day^−1^, *p* ≤ 0.05, d = 0.79). For BM, reductions (0.6 ± 0.4 kg) were observed post-activity (time effect: F_(1,8)_ = 25.78, *p* ≤ 0.05, partial ŋ2 = 0.763) that were comparable between trials (time x trial interaction: F_(1,8)_ = 0.067, *p* ≥ 0.05, partial ŋ2 = 0.008). Mean macronutrient intakes did not differ between conditions (CHO_TRAIN_: 2.3 ± 0.3 g·kg^−1^ BM, CHO_COMP_: 2.3 ± 0.5 g·kg^−1^ BM, *p* ≥ 0.05, d = 0.01, PRO_TRAIN_: 1.8 ± 0.4 g·kg^−1^ BM, PRO_COMP_: 1.4 ± 0.3 g·kg^−1^ BM, *p* ≥ 0.05, d = 1.26, FAT_TRAIN_: 38 ± 4.2% total EI, FAT_COMP_: 38 ± 4.1% total EI, *p* ≥ 0.05, d<0.01). A breakdown of activity-related EE for both activity scenarios can be seen in Table 4.

## 4. Discussion

The primary objective of this study was to provide an overview of EB within TRAIN and COMP scenarios by estimating TDEE and EI in international female R7s players. Energy deficits of −47% and −50% were observed throughout both TRAIN and COMP scenarios; findings that were confirmed in the sample of players completing both trials. Secondary findings highlighted that CHO intakes were lower than sports nutrition guidelines in both activity types (i.e., 60–62% of recommended values). PRO intakes exceeded the lower range of current sport nutrition recommendations in both scenarios (+42% and +25%, respectively). FAT intake within TRAIN was similar (FAT_TRAIN_: 35 ± 5% total EI) to the upper range of current sport nutrition guidelines (35% total EI) but exceeded the upper limit of recommendations in COMP (+11%, 39 ± 5% total EI). Accordingly, as optimal performance during competition preparation and the realization of training-induced adaptations are influenced by EB, female rugby R7s should consider their overall EB, together with the macronutrient composition of their EI, during both training and competition preparation scenarios.

Using an estimated method, this study highlighted that TDEE_TRAIN_ was comparable to elite male RU players (forwards: 15.9 ± 0.5 MJ·day^−1^, backs: 14 ± 0.5 MJ·day^−1^) [22] and female soccer players (11.7 ± 0.3 MJ) [7] during training weeks, whereas TDEE_COMP_ exceeded that of female touch rugby players (10.9 ± 0.8 MJ·day^−1^) [8] and female soccer players (12.2 ± 0.6 MJ) [7] reported previously. However, the EI of international female R7s players observed presently in both scenarios was lower than that reported in multi-match tournaments for elite netball (10.5 ± 3.8 MJ·day^−1^) [13] and touch rugby (10.0 ± 2.3 MJ·day^−1^) [8]. The implications of such findings throughout competition preparation might compromise performance during match-play [22], with long-term energy deficits resulting in a loss of BM and lean mass [32] as well as health-related consequences relating to the female athlete triad and/or relative energy deficiency in sport (RED-s) [4,5]. BM losses of 0.8 kg and 1.0 kg were observed in both TRAIN and COMP scenarios, respectively. Within-participant comparisons (*n* = 5) highlighted post-activity losses of 0.6 kg, which, although not statistically significant, demonstrated moderate ES. Such losses may be explained by the observed energy deficits seen between scenarios and/or potential sweat losses during each scenario. That said, the lack of difference between pre-training and pre-competition BM may be explained by the fact that participants may have entered into a phase of positive EB and hydration status between data collection phases. Although we observed mass losses over the assessment period, such data should be interpreted with caution given the cross-sectional design of this study. Accordingly, further longitudinal data would need to be ascertained to confirm longer-term health risks that may be associated with RED-s.

Despite not being able to assess FFM in either TRAIN or COMP scenarios, our descriptive findings (Table 5) indicate that relative kilocalories per kilogram of BM in international female R7s players are lower than the optimal threshold of ∼45 kcal·kg FFM^−1^·day^−1^ in both scenarios. The investigation indicates differences between EI and EE in an acute phase of training and competition preparation scenarios, and the potential for longer-term low energy availability (LEA) concerns (e.g., RED-s, etc.) warrants potential consideration. Loucks et al. [33] proposed that subclinical or reduced intakes of 30–45 kcal·kg FFM^−1^·day^−1^ may be undertaken over short periods or as part of a well-constructed weight-loss program [33]. The data obtained within the current investigation were undertaken over two separate, five-day periods; therefore, longer periods of energy expenditure and intake are required to substantiate the lower intakes of energy seen in the present study.

While a mismatch between EE and EI is often touted as a root cause of LEA and RED-s, it is unclear why certain athletes appear to be more likely to under-fuel and ultimately predispose themselves to LEA and RED-s [34]. It is proposed that disordered eating within athlete cohorts may underpin a large proportion of cases of LEA and typically occurs with increased prevalence among female athletes, with insufficient energy supply required to support athletic participation and training being a primary contributor for both RED-s and the Female Athlete Triad, LEA is viewed as a common metric of interest for both conditions [34]. Athletes may consciously or unconsciously lower their EI, either of which can increase the risk of LEA, particularly if this pattern continues over time [34]. The physiological effects, as well as the performance and health implications of RED-s, have been well studied [4,35,36,37], but a lack of research investigating LEA and RED-s in female RU exists. Disordered eating, eating disorders, and LEA may be of concern in female team sports. Sharps et al. [38] highlighted that of 112 female athletes screened, 53%, 44%, and 16% were at risk of LEA, disordered eating, and eating disorders, respectively. Of the 112 athletes, 44 listed RU as their primary sport, and whilst these findings cannot be directly extrapolated to the R7s variant of the game, these findings suggest a need to investigate such discrepancies over a longer timeframe to investigate LEA within female RU.

The observed CHO intakes may be sub-optimal to fully replete players’ glycogen reserves following rugby-specific activity, possibly compromising training and match-play performance and recovery [1,14], particularly where time is limited between repeated competitive encounters as per the playing format of R7s tournaments. Studies investigating CHO intake in RU have found wide-ranging values (i.e., 2.6–6.5 g·kg^−1^ BM; Black et al. [39]), with our findings being comparable to those observed previously [39] and lower than current recommendations when relativized to BM. Notably, Black et al. [39] suggested that the reduced CHO intakes relative to authoritative guidelines may reflect the practicalities of trying to achieve such intakes, as a 100+ kg player would be required to consume in excess of 600 g·day^−1^ of CHO when working towards the 6 g·kg^−1^·day^−1^ recommendations. Accordingly, while likely beneficial, achieving the intakes of published sports nutrition guidelines relative to BM may be challenging [39]. However, given the likely degradation of muscle glycogen concentrations in R7s training and competition, coupled with limited recovery periods between training and matches, promoting higher CHO intakes may be of benefit. Alternatively, in female athletes, lower intakes of CHO have been associated with athletes wishing to promote body composition changes [6], and this may also help to explain our findings. As within-player comparisons highlighted no differences in CHO intake between TRAIN and COMP, nutrition strategies that seek to increase CHO intake in international female R7s players should be considered with a view to optimizing performance throughout the competitive season.

R7s tournaments require multiple matches that incorporate repeated sprint activities performed with as little as three hours of recovery between competitive bouts [2]. Observations from other team sports indicate that reduced muscle glycogen concentrations result from match-play and are implicated in impaired high-intensity running and sprinting performance thereafter [40,41]. While the shorter duration of R7s match-play may limit the amount of glycogen depletion occurring compared to other team sports, it is notable that the highest rates of muscle glycogen depletion occur in the first half of such events [41]. Given the match-play demands of international R7s, coupled with sub-optimal energy and CHO intakes observed within this study, it is possible that muscle glycogen concentrations may be compromised; particularly if numerous matches are played in a short timeframe. Notably, Bradley et al. [40] reported no differences in the movement patterns or pre-match muscle glycogen concentrations when Rugby League players consumed either a low/moderate (i.e., 3 g·kg^−1^·day^−1^) or moderate/high (i.e., 6 g·kg^−1^·day^−1^) CHO diet in the 36 h preceding match-play. That said, differences in the anthropometric characteristics of the participants in these studies versus the cohort recruited for our study mean that caution should be exercised when comparing findings. Therefore, while no studies have investigated changes in muscle glycogen concentrations during single, or repeated, bouts of R7s training or match-play, it is unclear whether the effects of nutritional intakes observed here are optimal for performance and recovery; a statement that may be pertinent when seeking to enhance R7s performance.

Lower PRO intakes observed during COMP versus TRAIN may be due to limited or inappropriate food choices when on tour and/or during competition [2]. Sub-optimal choices during COMP are further supported by player feedback to the lead researcher during the face-to-face consultation ascertaining the accuracy of the COMP food diaries. Given the EI findings presented in this study, daily PRO intake (TRAIN: 1.7 ± 0.4 g·kg^−1^ BM, COMP: 1.5 ± 0.1 g·kg^−1^ BM) was meeting the minimum sports nutrition recommendations. However, as higher daily PRO intakes of 1.8–2.7 g·kg^−1^ BM have been reported during periods of energy deficit [42], and within participant comparison indicated the lower end of this range was not being achieved during competition preparation (1.4 ± 0.3 g·kg^−1^ BM), it may be pertinent to comment that nutrition strategies for international female R7s players should seek to increase PRO intake while also increasing CHO intake to optimize performance and maximize training adaptations to facilitate optimal performance during competition preparation and subsequent tournament scenarios.

Total daily FAT intake in TRAIN exceeded current authoritative recommendations [15]. During COMP, FAT intake exceeded recommendations (20–35% total EI) [15] and was comparable to that previously reported (36 ± 5% total EI) in female volleyball players [13]. These findings are further supported by within-player comparisons between conditions that highlighted that total FAT intake exceeded current recommendations in both scenarios [15]. Higher FAT intakes during competition preparation may be associated with limited or inappropriate food choices at venues during preparation and competitive tournaments [2], and these may be contributing factors to explaining our findings. Given these findings, manipulation of macronutrient intakes, to facilitate a reduced overall EI from FAT with increased CHO, may be warranted and requires further investigation.

Micronutrient analysis within both TRAIN and COMP scenarios indicated that female R7s players consumed higher dietary intakes of calcium and iodine in TRAIN and higher dietary intakes of selenium and vitamin C in COMP than current Scientific Advisory Committee on Nutrition (SACN) recommendations. Both vitamins A and E have higher dietary intakes in both scenarios. Calcium is a known mediator of energy metabolism and muscle contraction [43,44], and despite consumption being higher than published guidelines, intakes of calcium were lower than recommended for athletes by organizations such as the IOC (1500 mg·day^−1^) [15,45]. Given the competition and match-play demands of R7s, it may be pertinent for sport nutrition practitioners to consider calcium intakes in relation to muscle contraction activities and potential recovery modalities [46,47] in female R7s players, which warrants further investigation within this cohort relating to optimal daily requirements to elicit these adaptations in both training and match-play scenarios. Similarly, despite vitamin E being regarded as an antioxidant that reduces exercise-induced reactive oxygen species and supports immune function [45], intakes within female R7s players were higher than SACN recommendations for vitamin E; however, there is limited evidence to support higher daily intakes in relation to exercise recovery [45,48], with some research suggesting higher intakes may be detrimental to immune system function [45,48]. As our findings suggest a 97% elevated intake of vitamin E compared to SACN recommendations and evidence suggests higher intakes of vitamin E may not be beneficial to athletes, these findings provide an opportunity to improve athlete education regarding appropriate intakes of dietary sources.

Interestingly, iron intakes were lower ~27% than both SACN recommendations (14.8 mg·d^−1^) and sports nutrition recommendations with regards to daily intakes for athletes who may be deficient (≥18 mg·d^−1^) [15,45]. Despite a lack of statistical significance, mean iron takes during COMP were also lower than SACN recommendations (COMP: 12.4 ± 3.2 mg vs. SACN: 14.8 mg·d^−1^). Given the associated roles of dietary iron within exercise physiology (e.g., oxygen transfer, red blood cell formation, etc.), roles that, in female athlete cohorts, become of greater importance given often lower serum values due to menstrual cycles [45]. Such intake values may be associated with athlete food choices within these scenarios, offering sport science practitioners insight into (a) dietary iron intakes within international female R7s players within a TRAIN and COMP scenario and (b) an opportunity to investigate avenues of physiological adaptations in relation to oxygen kinetics and optimize dietary iron intakes within female R7s cohorts with a view to improving such adaptations and performance.

To the authors’ best knowledge, this is the first study to estimate EE and EI within international female R7s players. However, this study is not without its limitations. Firstly, due to competition regulations, wrist-worn devices during competitive matches are strictly prohibited, and in keeping with the practices of the team, devices were not worn during the tournament competition days. This is an important consideration when considering nutritional strategies in such environments; however, research in this field using such devices during competition is limited. Walker et al. [49] used inertial sensors to estimate EE in Australian football (training: 2719 ± 666 kJ, matches: 5745 ± 1468 kJ); however, with the contacts/collisions involved within training/matches, it may be possible that the additional EE involved with these types of game-specific movements is underestimated by wearable technology [50]. This is further supported by Costello et al. [19], who demonstrated that EE was higher during a five-day training cycle containing collision-based activities (95.07 ± 16.66 MJ) compared to a non-collision training cycle (90.34 ± 16.97 MJ). These are important findings in relation to athlete EE in collision sports, and future research could be guided by using these methodologies for female athletes within collision sports. Acknowledging this, it may be possible that our findings underestimate EE in international female R7s players during training and competition scenarios as collision-based activities took place in both scenarios. Lastly, it must be recognized that despite offering some insight into the estimated EE and EI practices of international female R7s players, the sample is taken from a single National team, with data collected at a specific timepoint within a training and competition cycle.

Validated self-reported food diaries were used as a method of estimating EI in a manner that agreed with the practices of the team [28], but these methods may be subject to participant under-reporting [6]. To negate the potential of under-reporting, it has been proposed that a minimum level of EE that equates to 1.1 × BMR can be used as a threshold to represent true habitual intake [29]. In this study, it is possible that underreporting accounted for differences observed in EI and EE as reported intake was below this threshold in both training and competition scenarios, with empirical evidence supporting our findings. Future studies may wish to consider additional methods of assessing dietary intake, such as weighed FD or additional dietary recall to validate EI within the FD.

## 5. Conclusions

Acknowledging the potential limitations of using estimated means of EE and EI, these findings indicate that EE exceeded EI in both training and competition preparation scenarios in international-standard female R7s players. CHO and FAT intakes in both activity types were sub-optimal when compared to the current sports nutrition recommendations, findings that may have implications for the optimized realization of training adaptations and performance. Further research into TDEE and EI within intermittent team sports within applied settings, particularly in female cohorts, is warranted to support the findings of this study. Notably, researchers working within such populations may wish to consider alternative methods of EE measurement (e.g., DLW, direct calorimetry, etc.) to substantiate our findings. Moreover, all methods of estimating EI are not without their limitations, but researchers may wish to adopt differing applied methods of EI that decrease participant burden while enabling the accuracy of EI within an applied, team-sport setting. Practitioners should also consider both competition and training demands when seeking to optimize nutritional strategies within elite female R7s. A focus on player education to allow for the manipulation of macronutrients, particularly the reduction in the total EI percentage of fat and increased consumption of CHO in line with current authoritative guidelines, may be of benefit.

## Figures and Tables

**Table 1:** Overview of the training (*n* = 5 days) and competition preparation (*n* = 5 days) week schedule.

	Training Week				
	Day 1	Day 2	Day 3	Day 4	Day 5
a.m.	Prehabilitation Gym	Prehabilitation Rugby training	Active recovery	Gym	Prehabilitation Rugby training
p.m.	Rugby training	Gym		Prehabilitation Rugby training	Gym
	**Competition Preparation Week**				
	**Day 1**	**Day 2**	**Day 3**	**Day 4**	**Day 5**
a.m.	Active recovery	Prehabilitation Rugby training	Prehabilitation Rugby training	Active recovery	Prehabilitation Rugby training
p.m.	Rugby training	Gym			Gym

**Table 2:** Mean (± standard deviation) daily macronutrient intakes compared to current sports nutrition recommendations (Thomas et al. [15] and Phillips and Van Loon [31]).

	Training				Competition			
	CHO	PRO	FAT	FAT	CHO	PRO	FAT	FAT
	(g.kg^−1^ BM)	(g.kg^−1^ BM)	(g.kg^−1^ BM)	(% total EI)	(g.kg^−1^ BM)	(g.kg^−1^ BM)	(g.kg^−1^ BM)	(% total EI)
Day 1	2.2 ± 0.5	1.9 ± 0.6	1.0 ± 0.5	35.0 ± 8.3	2.2 ± 1.1	1.4 ± 0.5	1.0 ± 0.4	39.1 ± 8.2
Day 2	2.4 ± 0.7	1.7 ± 0.5	1.1 ± 0.4	34.9 ± 9.0	2.5 ± 0.6	1.7 ± 0.5	1.1 ± 0.2	38.2 ± 4.3
Day 3	2.3 ± 0.7	1.3 ± 0.3	1.0 ± 0.5	37.4 ± 9.9	2.5 ± 0.7	1.3 ± 0.5	1.1 ± 0.4	38.3 ± 4.9
Day 4	2.5 ± 0.8	2.1 ± 0.8	1.0 ± 0.3	32.6 ± 5.0	2.6 ± 1.4	1.7 ± 0.5	1.3 ± 0.4	41.0 ± 7.5
Day 5	2.4 ± 0.4	1.8 ± 0.4	1.0 ± 0.3	35.0 ± 5.4	2.4 ± 0.6	1.6 ± 0.3	1.0 ± 0.3	36.5 ± 8.9
**Recommendation**	**6–10**	**1.2–2.0**	**N/A**	**20–35**	**6–10**	**1.2–2.0**	**N/A**	**20–35**

**Table 3:** Mean (± standard deviation) daily macronutrient intakes compared to Scientific Advisory Committee on Nutrition (SACN) daily intake recommendations in a training (TRAIN) and competition preparation (COMP) scenario.

	Micronutrient									
	Sodium (mg)	Potassium (mg)	Calcium (mg)	Iron (mg)	Selenium (mg)	Iodine (mg)	Vitamin A (µg)	Vitamin C (mg)	Vitamin D (µg)	Vitamin E (mg)
TRAIN	1716 ± 458	2980 ± 463	1047 ± 323	11.3 ± 2.3	63.4 ± 15.4	205 ± 83	857 ± 282	90 ± 81	6.8 ± 9.2	8.7 ± 3.1
%diff	7	−16	40	−27	6	38	35	77	38	97
COMP	1850 ± 604	3628 ± 837	847 ± 276	12.4 ± 3.2	77 ± 17	164 ± 50	1037 ± 269	115 ± 69	8.6 ± 3.9	11 ± 2.1
%diff	14	4	19	−18	25	16	53	97	15	114
**SACN**	**1600**	**3500**	**700**	**14.8**	**60**	**140**	**600**	**40**	**10**	**3**

%diff = percentage difference from Scientific Advisory Committee on Nutrition (SACN) daily intake recommendations.

**Table 4:** Mean (± standard deviation) estimated activity energy expenditure (MJ·day^−1^) of international female rugby sevens players (*n* = 5) in a training and competition preparation scenario.

	Training					Competition Preparation				
Day	Prehabilitation	Gym Training	Rugby Training	Active Recovery	Non-Activity Related EE	Prehabilitation	Gym Training	Rugby Training	Active Recovery	Non-Activity Related EE
1	2.1 ± 0.6	1.9 ± 0.4	2.7 ± 0.5		3.9 ± 1.1			1.8 ± 0.5	2.7 ± 0.4	5.4 ± 1.2
2	1.3 ± 0.5	1.3 ± 0.3	2.6 ± 0.3		4.7 ± 1.9	1.5 ± 0.4	1.3 ± 0.2	1.8 ± 0.6		4.4 ± 1.1
3				1.7 ± 0.3	5.8 ± 1.6	1.4 ± 0.4	1.4 ± 0.8			5.0 ± 1.2
4	0.4 ± 0.2	2.1 ± 0.4	1.3 ± 0.5		4.1 ± 0.6				2.3 ± 0.3	6.4 ± 0.8
5	1.1 ± 0.1	1.0 ± 0.1	2.1 ± 0.3		5.0 ± 0.9	1.1 ± 0.5		1.6 ± 0.3		5.5 ± 1.0

**Table 5:** Relative kilocalories per kilogram of body mass (kcal∙BM^−1^) in both training (TRAIN; *n* = 11) and competition (COMP; *n* = 8) preparation of international female rugby sevens players.

	kcal∙BM^−1^											
Player	Player 1	Player 2	Player 3	Player 4	Player 5	Player 6	Player 7	Player 8	Player 9	Player 10	Player 11	**Mean**
TRAIN (n = 11)	22.6	23.9	33.9	24.7	26.6	29.6	24.1	25.4	27.0	22.6	28.1	**25.8 ± 4.2**
COMP (n = 8)	23.7	23.7	30.9	21.9	21.4	28.6	32.3	23.6	n/a	n/a	n/a	**26.2 ± 3.4**

## Data Availability

The data presented in this study are available on request from the corresponding author.
